# Looking for A Place for Dose-Dense TMZ Regimens in GBM Patients: An Experience with MGMT Exploratory Evaluation

**DOI:** 10.3390/bioengineering6010011

**Published:** 2019-01-22

**Authors:** Luca Napoleoni, Alessio Cortellini, Katia Cannita, Alessandro Parisi, Antonella Dal Mas, Giuseppe Calvisi, Olga Venditti, Paola Lanfiuti Baldi, Valentina Cocciolone, Alessandro Ricci, Corrado Ficorella

**Affiliations:** 1 Medical Oncology, St Salvatore Hospital, 67100 L’Aquila, Italy; lucanapoleoni@hotmail.it (L.N.); kcannita@gmail.com (K.C.); alexparis@hotmail.it (A.P.); olgavenditti@yahoo.it (O.V.); paola.lb@tiscali.it (P.L.B.); corrado.ficorella@univaq.it (C.F.); 2 Department of Biotechnological and Applied Clinical Sciences, University of L’Aquila, 67100 L’Aquila, Italy; valentinacocciolone@gmail.com; 3 Department of Pathology, St Salvatore Hospital, 67100 L’Aquila, Italy; antonelladalmas@yahoo.it (A.D.M.); t.calvisi@libero.it (G.C.); 4 Department of Neurosurgery, St Salvatore Hospital, 67100 L’Aquila, Italy; aricci@asl1abruzzo.it

**Keywords:** glioblastoma multiforme, dose-dense, temozolomide, MGMT

## Abstract

Prolonged exposure to temozolomide (TMZ) could improve clinical outcomes in recurrent glioblastoma multiforme (GBM) patients. We previously developed a dose-dense regimen of TMZ in a phase II study (180 mg/m^2^ from days 1 to 5 every two weeks). A retrospective analysis of patients with macroscopic residual GBM treated with “post-induction” dose-dense TMZ was conducted, adding an explorative subgroup analyses among patients with different *O*^6^-methylguanine DNA methyltransferase (MGMT) expressions (negative vs positive, < vs ≥ of 50 % of cells stained, < vs ≥ 70% of cells stained). Thirty-six patients were evaluated; after a median follow-up of 36 weeks, median Progression Free Survival (PFS) and median Overall Survival (OS) were 19 and 34 weeks, respectively. MGMT expression (70% cut-off) and sex were confirmed as independent predictors for disease control rate (DCR) at multivariate analysis. At univariate analysis ECOG-PS, Sex (female), extensive tumor resection was shown to be related to a longer PFS, while MGMT expression (cut-off 70%) to a shorter PFS. Multivariate analysis with Cox hazard regression confirmed only ECOG-PS as an independent predictor for PFS. ECOG-PS showed to be significant related to a longer OS. Our analysis showed that dose-dense TMZ regimens are still an option for patients with recurrent GBM, but should be used for re-challenge treatments. MGMT immunohistochemistry high expression might be used as a “surrogate” negative predictor for DCR for dd-TMZ treatments.

## 1. Introduction

The current standard of care for patients with newly diagnosed glioblastoma multiforme (GBM) is maximum safe surgical resection followed by concomitant temozolomide (TMZ) and radiation therapy (RT), and then adjuvant TMZ (5/28 day cycle), based on the results of the Stupp clinical trial [1]. The cytotoxic effect of TMZ primarily depends on transferring a methyl group to the *O*^6^ position of guanine, generating DNA lesions, and subsequently, apoptosis [2]. *O*^6^-methylguanine DNA methyltransferase (MGMT) is a DNA repair enzyme that has been established as a major mechanism of resistance to alkylating agents, such as TMZ [3]. It transfers methyl adducts from the *O*^6^ position of guanine residue to its cysteine, in its active enzymatic domain, but this action in turn leads to inactivation and consumption of MGMT itself, and subsequent degradation; therefore, high MGMT expression levels in cells may confer resistance to TMZ. Methylation of the promoter region of the *MGMT* gene cause silencing of MGMT enzyme expression and has been associated with a statistically significant improvement in progression free survival (PFS) and overall survival (OS) in patients receiving combined radiotherapy and TMZ regimen [4]. These findings suggest that modulation of MGMT enzymatic activity may increase sensitivity and response to TMZ regimens; indeed, a prolonged exposure to alkylating agents has been shown to deplete intracellular MGMT in peripheral blood mononuclear cells [5]. 

Different “dose-dense” (dd) TMZ regimens (7/14 d, 21/28 d, 28/28 d regimen) were studied in patients with recurrent or progressive GBM, previously exposed to TMZ, to reduce MGMT levels, particularly in MGMT un-methylated GBM patients [6]. Many phase II trials have demonstrated the feasibility of dd-TMZ regimens in the recurrent setting, supporting the hypothesis that a re-challenge with a dd-TMZ regimen could be beneficial to recurrent or progressive tumors, which have evaded previous exposure to the standard dose of TMZ [7,8,9,10,11,12,13,14]. On the other hand, a randomized phase III clinical trial compared the standard adjuvant TMZ treatment with dd-TMZ (21/28 day cycle) in newly diagnosed GBM, showing no improvement in survival [15].

In order to evaluate the feasibility of an alternative dd-TMZ regimen, we previously developed a phase II study, with a dose-finding initial cohort, in patients with high grade gliomas (World Health Organization grade 3 and 4) [16]; our clinical experience with “dose-dense” dd-TMZ involved 70 patients until December 2016. 

Here we report a retrospective analysis of patients with macroscopic residual GBM after surgery, treated with “post-induction” dd-TMZ.

## 2. Materials and Methods

### 2.1. Dose Finding Cohort

The dose-finding cohort was designed to evaluate maximum tolerated dose (MTD), and recommended dose of TMZ, for subsequent clinical development of the regimen. It was planned according to an intra- and inter-patient titration [17,18], with seven dose levels. DLT (dose limiting toxicity) was defined as hematological grade 4, non-hematological grade 3, and any toxicity requiring >2 week treatment delay. From October 2002 to November 2008, 13 patients were enrolled in the dose-finding cohort. MTD was reached at the V dose level, and recommended dose resulted the IV level (180 mg/m^2^ from days 1 to 5 every two weeks).

### 2.2. Patient Eligibility

In the subsequent clinical development of dd-TMZ regimen, further 57 patients were treated at our institution. In the present retrospective analysis, we evaluated only patients With after-surgery macroscopic residual GMB, treated with “post-induction” dd-TMZ regimen, after previous concomitant TMZ/RT. All patients had histologically confirmed diagnosis of GBM, according to WHO central nervous system (CNS) tumor classification [19,20]. Four out of 36 patients were enrolled in the dose-finding cohort. Patients were candidate to receive dd-TMZ if they fit the following criterion: age 18–75 years, Eastern Cooperative Oncology Group PS (ECOG-PS) ≤ 2, adequate haematological, renal, and hepatic functions. All patients provided written informed consent to the proposed treatment and to participate to this analysis (those alive at the moment of data collection). All the available procedures to ensure anonymity were used to guarantee the confidentiality of personal information of deceased patients. The procedures followed were in accordance with the precepts of Good Clinical Practice and the ethical standards of local responsible committee on human experimentation (Comitato Etico per le province di L’Aquila e Teramo). 

### 2.3. Treatment Schedule 

Dd-TMZ regimen is an intensive schedule of oral temozolomide, administered at the recommended dose of 180 mg/m^2^, from day 1 to day 5, with cycles of 14 days. Four patients were treated in the dose-finding cohort: from 150 to 200 mg/m^2^. Previous concomitant TMZ/RT consisted of the conventional regimen of TMZ with fractionated focal irradiation at dose of 2 Gy per fraction, given once daily five days a week (from Monday to Friday), over a period of six weeks (total dose of 60 Gy). Radiotherapy was delivered to the gross tumor volume with a 2-to-3-cm margin for the clinical target volume.

### 2.4. MGMT Analysis

MGMT immunohistochemistry assay was retrospectively performed on formalin-fixed, paraffin-embedded sections, which were deparaffinised in xylene and rehydrated in increasing concentrations of ethanol. After blocking of endogenous peroxidase with 3% H_2_O_2_, the sections were pre-treated in an oven with EnVision Flex TRS buffer and immunostained on a DAKO Cytomation autostainer (DAKO, Glostrup, Denmark), using monoclonal mouse anti-human antibody against MGMT (clone MT3.1, MAB16200 EMD Millipore^TM^, Billerica, MA, USA). Immunoreactivity was visualized with DAB+ (DAKO K3468) as chromogen. The immunohistochemical reactions were semiquantitatively evaluated according to the number of tumor cells stained. Subgroup analyses were exploratory performed using three cut-off of MGMT expression: negative vs positive, < vs ≥ of 50% of cells stained, and < vs ≥ 70% of cells stained. 

### 2.5. Study Design and Statistical Analysis

A retrospective analysis of patients with after-surgery macroscopic residual GBM, treated with “post-induction” dd-TMZ was conducted. After-surgery macroscopic residual GBM consisted of a measurable post-operative residual disease; these patients were treated with “induction” concomitant TMZ/RT, followed by “post-induction” dd-TMZ regimen. Clinical evaluation of response was performed by magnetic resonance imaging (MRI) in clinical practice through the years, according to clinician preference. Follow-up was scheduled every three months, up to progression or death. Objective response rate (ORR) was defined as the portion of patients experiencing an objective response (complete response, CR, or partial response, PR) as best response; disease control rate (DCR) was defined as the portion of patients which experienced an objective response or demonstrated stable disease (SD) as best response. PFS was defined as the length of time from dd-TMZ commencement and disease progression or death (resulting from any cause) or to the last contact; OS was defined as the length of time from dd-TMZ commencement and death (or last contact). ORR and DCR were evaluated according to RECIST criteria (Version 1.0 before 2010 and version 1.1 subsequently) [21,22]. Toxicity was registered according to National Cancer Institute Common Toxicity Criteria (version 3.0 before 2009 and version 4.0 subsequently). The data cut-off period was January 2018. Median PFS and median OS were evaluated using the Kaplan–Meier method [23]. The following covariates were analyzed: extensive vs non-extensive tumor resection (in case or not the surgery was performed with a radical intent) [24], elderly vs non-elderly patients (>64 years old) [25], ECOG-PS 0/1 vs 2, sex (male vs female), and MGMT expression (according to the abovementioned cut-offs). Fisher’s exact test [26] was used to compare DCR among subgroups (subgroup analyses were not performed for ORR). Cox proportional hazards model [27] was used to evaluate predictor variables. Multiple regression was used to evaluate the role of parameters, which was shown to be significantly related to DCR [28]. All the statistical analyses were performed with MedCalc Statistical Software, version 16.4.3 (MedCalc Software bvba, Ostend, Belgium; https://medcalc.org; 2016). 

## 3. Results

### 3.1. Patients’ Features

From February 2006 to December 2016, 36 consecutive patients, with after-surgery macroscopic residual GBM, were treated with “post-induction” dd-TMZ regimen (4 previously enrolled in the dose-finding cohort, and 32 subsequently). Clinical features of patients are summarized in Table 1. Male/Female ratio was 23/13, and median age was 61 years. Thirteen (36.1%) patients were elderly and 23 (66.2%) patients were non-elderly. Twenty-eight (77.1%) patients had ECOG-PS 0–1, while eight (22.2%) patients had ECOG-PS 2. Nineteen patients (52.8%) underwent an extensive tumor resection and 17 (47.2%) a non-extensive/surgical biopsies. All patients were evaluable for MGMT expression, distinguished as follows: 7 (19.4%) negative and 29 (80.6%) positive, 12 (33.3%) ≥ 50% and 24 (66.7) < 50%, 7 (19.4%) ≥ 70% and 29 (80.6%) < 70%. 

### 3.2. Activity and Efficacy

**Figure 1:**
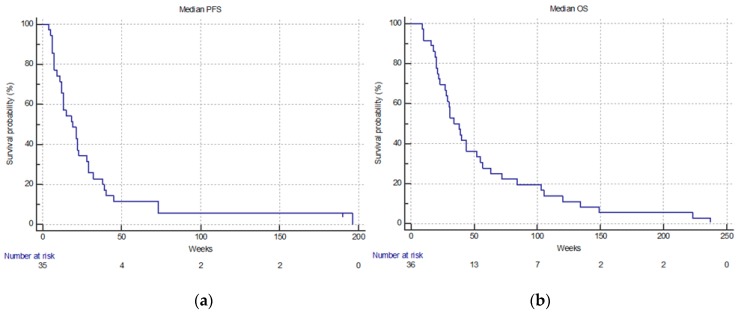
Kaplan–Meier survival estimate; overall treated patients. (**a**) Median progression-free survival: 19.0 weeks (95% CI: 12–28); (**b**) Median overall survival: 34.0 weeks (95% CI: 28–52).

All patients were evaluable (Table 2). ORR was 19.4% (1 CR and 6 PR) and DCR was 55.5% (13 SD). After a median follow-up of 36 weeks (range: 9–237) median PFS was 19 weeks (95% CI: 12–28) and median OS was 34 weeks (95% CI: 28–52) (Figure 1). 

As Table 3 shows, at univariate analysis sex (in favor of female), ECOG-PS (in favor of PS 0–1), and MGMT expression (70% cut-off, in favor of <70% of staining) were significantly related to DCR. At multivariate analysis, MGMT expression (cut-off 70%) and sex were confirmed as independent predictors for DCR. 

At univariate analysis ECOG-PS (0/1 vs ≥ 2), sex (female vs male) and extensive tumor resection showed to be significantly related to a longer PFS, while MGMT expression ≥70% to a shorter PFS; multivariate analysis only ECOG-PS was confirmed as an independent predictor for PFS. At univariate analysis, ECOG-PS 0/1 and extensive tumor resection showed to be significantly related to a longer OS; only ECOG-PS was confirmed at multivariate analysis (Table 4).

### 3.3. Dose-Intensity 

Among overall patients, the median number of administered cycles was 5.5 (range: 1–46) and “per cycle” median received dose intensity (rDI) was 168.3 mg/m^2^ (93.5% of projected DI). 

### 3.4. Toxicity

The most relevant grade 2 toxicities were leucopenia (25%), transaminases increase (16.7%), neutropenia (11.1%), constipation (11,1%), thrombocytopenia (11.1%), and asthenia (13.9%). Among grade 3 adverse events, anemia was 2.8%, leucopenia was 11.1%, neutropenia was 8.3%, and thrombocytopenia was 5,6%. Among grade 4 adverse events, leucopenia was 2.8%, neutropenia was 2.8%, and thrombocytopenia was 5,6%. Grade 3/4 non hematological toxicities were not reported. No death was suspected to be related to an adverse event (Table 5).

### 3.5. Subsequent Treatments

Thirteen patients (36.1%) who progressed to/after “post-induction” dd-TMZ underwent a second line chemotherapy. Irinotecan was used in eight patients (61.5%), fotemustine was used in three patients (23.1%) and fotemustine-bevacizumab association was used in two patients (15.4%). One patient underwent a third line chemotherapy with Carboplatin.

## 4. Discussion

It is now known that prolonging exposure to TMZ can improve clinical outcomes over other alkylating agents in recurrent GBM [6]. To find an appropriate location in the therapeutic algorithm for our dd-TMZ regimen, this analysis was focused on patients with after-surgery macroscopic residual disease. Despite the limitations of the sample size and the retrospective nature, we can say that the clinical outcomes are quite comparable to other experiences with dd-TMZ [7,8,9,10,11,12,13,14], and the safety profile overlaps with both the current standard of care and other intensified regimes. In our opinion, dd-TMZ regimen should not be used as adjuvant treatment for newly diagnosed GBM patients, who are TMZ-naïve. However, a re-challenge with a dd-TMZ schedule could be used in recurrent or progressive tumors, particularly in case of progressive disease after completion of adjuvant treatment (with or without a treatment-free interval). In support of the prolonged used of TMZ, a standard therapy in second-line setting has not yet been established [29]; notably 36.1% of our patient underwent a further line therapy. *MGMT* gene inactivation due to promoter methylation may have a positive predictive role for treatment with TMZ and eventually correlate with a better prognosis [3,4,30].

Immunohistochemistry evaluation of MGMT can be easily performed on archival paraffin-embedded tissue, so it might be taken into account as a “surrogate assessment” of MGMT promoter methylation. Moreover, promoter methylation is not the only factor involved in regulation of MGMT function [31,32,33,34,35]. Nevertheless, correlation between MGMT protein expression, assessed by immunohistochemistry staining, and MGMT promoter methylation in gliomas is not uniquely established, so we must take with caution our results, recognizing that immunohistochemistry is not a reliable method that can guide the decision-making process in clinical practice.

Our explorative subgroup analysis among three levels of MGMT expression revealed interesting results; only the expression cut-off of ≥ 70% was confirmed as an independent predictor for a worse DCR. If it is true that MGMT expression has a negative predictive role for the treatment with TMZ, and that immunohistochemistry staining is a continuous variable, we are allowed to think that the more "intense" the dose is, the higher the expression cut-off (shown to be a significant predictor) is. With this in mind, it is noticeable that the significant cut-off is the highest one among the three exploratory levels. 

While considering all the efforts in establishing the predictive role of MGMT for TMZ-based treatment, we must to face off with the lack of therapeutic options for patients with un-methylated tumors. In referring to the abovementioned, in our opinion, immunohistochemistry MGMT evaluation is not reliable enough to guide the decision-making process, even more in avoiding TMZ-based treatments in patients with very high expression (≥70%). Surely, the methylation test could be more reliable, but to our knowledge there are no other available options in clinical practice (out of clinical trial), and TMZ-based treatment might be avoidable only in older patients with un-methylated MGMT [36].

Our results of a better trend in clinical outcomes in female patients are not conclusive. We can take them as an opportunity to reflect on sex-driven differences in cancer biology and immunity, in brain tumors particularly [37]. Regarding extensive and non-extensive resection, even if our results at multivariate analyses of a better PFS and OS seem not to be in agreement with literature, surgical resection of bulky disease, even at recurrence, should always be taken into consideration [38]. 

For a comprehensive discussion, we must consider the historical lack of therapeutic options for recurrent GBM patients. Novel approaches, such as gene therapy and immunotherapy, are still far from becoming common clinical practice [39,40]; therefore, forcing the use of the same agent and intensifying its dose has always been considered the “last bad choice”.

## 5. Conclusions

Dose-dense TMZ regimens are still an option for patients with recurrent GBM; our experience seems to be aligned to previously published results. In our current clinical practice, dd-TMZ was used for re-challenge treatments, while conventional TMZ regimen was used for TMZ-naïve patients (including newly diagnosed GBM with after-surgery macroscopic residual disease). MGMT immunohistochemistry high expression (cut-off ≥ 70%), besides being easy to perform, may be used as a “surrogate” negative predictor for treatment with dd-TMZ, even if its validation will remain a matter of debate.

## Figures and Tables

**Table 1:** Patients’ features.

No. of Patients (%), Total 36	
Gender	
Male	23 (63.2)
Female	13 (36.1)
Age (Years)	
Median	61
Range	(24–75)
Elderly (≥65)	13 (36.1)
ECOG-PS	
0–1	28 (77.8)
2	8 (22.2)
Type of Surgery	
Extensive resection	19 (52.8)
Non–extensive resection	17 (47.2)
MGMT (IHC Expression)	
Positive	29 (80.6)
Negative	7 (19.4)

**Table 2:** Activity and efficacy data.

	Overall	
	N°	%
Evaluable Patients	36	100
Objective Response Rate	19.4% (95% CI: 7.8–40.1)	
Partial Response	6	
Complete Response	1	
Disease Control Rate	55.5% (95% CI: 33.9–85.8)	
Stable Disease	13	
Progression Disease	16	
Median PFS (weeks)	19.0	
Range	4–196	
Progression events	35	
Median OS (weeks)	34	
Range	9–237	
Deaths	35	

**Table 3:** Univariate analysis with Fisher exact test (binomial confidence interval) and multivariate analysis with multiple regression. *: statistically significant.

Univariate Analysis			
Variable (N°)	DCR (95% CI)		*p*-value
Age			
Non Elderly (23)	56.5% (34.4–76.8)		1.0000
Elderly (13)	53.8 % (25.1–80.7)		
ECOG-PS			
0–1 (28)	67.9% (47.6–84.1)		0.0121 *
2 (8)	12.5% (0.3–52.6)		
Sex			
Male (23)	39.1% (19.7–61.4)		0.0139 *
Female (13)	84.6% (54.5–98.1)		
Type of resection			
Extensive (19)	68.4 % (43.4–87.4)		0.1786
Non-extensive (17)	41.2% (18.4–67.1)		
MGMT expression			
Positive (28)	51.7% (32.5–70.5)		0.4264
Negative (7)	71.4% (29.1–96.3)		
MGMT expression			
≥50% (12)	41.7% (15.1–72.3)		0.2983
<50% (24)	62.5% (40.5–81.2)		
MGMT expression			
≥70% (7)	14.3% (0.3–57.8)		0.0298 *
<70% (29)	65.5% (45.6–82.1)		
Multivariate Analysis			
Variable	Coefficient	Std. Error	*p*-value
ECOG-PS 0–1 vs 2	0.3482	0.1712	0.0503
Sex male vs female	0.3630	0.1481	0.0199 *
MGMT (70% cut-off)	0.5153	0.1650	0.0038 *
Coefficient of Determination R^2^: 0.4567			

**Table 4:** Univariate and multivariate Cox hazard regression analysis for PFS and OS. *: statistically significant.

Univariate Analysis				
Variable (N°)	PFS		OS	
	HR (95% CI)	*p*-value	HR (95% CI)	*p*-value
Age				
Non Elderly (23) Elderly (13)	1.3 (0.6–2.7)	0.4068	1.7 (0.8–3.5)	0.1526
ECOG-PS				
0–1 (28) 2 (8)	26.3 (5.2–132.4)	0.0001 *	5.1 (2.1–12.3)	0.0003 *
Sex				
Male (23) Female (13)	2.2 (1.1–4.5)	0.0328 *	1.2 (0.6–2.4)	0.5828
Type of resection				
Extensive (19) Non-extensive (17)	3.2 (1.5–7.0)	0.0028 *	2.7 (1.3–5.6)	0.0058 *
MGMT expression				
Positive (28) Negative (7)	1.6 (0.6–4.3)	0.3251	1.2 (0.5–2.7)	0.7209
MGMT expression				
≥ 50% (12) < 50% (24)	1.2 (0.6–2.4)	0.6838	0.9 (0.4–1.9)	0.8945
MGMT expression				
≥ 70% (7) < 70% (29)	2.7 (1.1–6.9)	0.0379 *	2.1 (0.9–5.2)	0.0803
Multivariate Analysis				
ECOG-PS	11.5 (1.9–66.9)	0.0064 *	3.3 (1.2–9.1)	0.0179 *
Type of resection	2.1 (0.8–5.3)	0.0898	1.9 (0.8–4.5)	0.1228
MGMT (cut-off 70%)	2.5 (1.01–6.38)	0.0592	-	-
Sex	2.54 (0.9–6.6)	0.1507	-	-

**Table 5:** “Per patient” toxicity data.

Number	Overall Patients			
NCI-CTC Grade	1	2	3	4
Asthenia (%)	17 (47.2)	5 (13.9)	-	-
Anorexia (%)	4 (11.1)	-	-	-
Nausea (%)	7 (19.4)	1 (2.8)	-	-
Vomiting (%)	3 (8.3)	1 (2.8)	-	-
Dysgeusia (%)	3 (8.3)	1 (2.8)	-	-
Mucositis (%)	1 (2.8)	-	-	-
Constipation (%)	12 (33.3)	4 (11.1)	-	-
Rash (%)	1 (2.8)	1 (2.8)	-	-
Increased transaminases (%)	17 (47.2)	6 (16.7)	-	-
Edema (%)	7 (19.4)	2 (5.6)	-	-
Anemia (%)	5 (13.9)	3 (8.3)	1 (2.8)	-
Leukopenia (%)	12 (33.3)	9 (25)	4 (11.1)	1 (2.8)
Neutropenia (%)	7 (19.4)	4 (11.1)	3 (8.3)	1 (2.8)
Thrombocytopenia (%)	9 925)	4 (11.1)	2 (5.6)	2 (5.6)
